# Severe reaction in a child with asymptomatic codfish allergy: Food challenge reactivating recurrent pancreatitis

**DOI:** 10.1186/1824-7288-38-16

**Published:** 2012-05-09

**Authors:** Katia Pellegrino, Leila Emma D’Urbano, Maria Cristina Artesani, Carla Riccardi, Sandro Mancini, Sergio Bella, Federico Alghisi, Giovanni Cavagni

**Affiliations:** 1 Department of Paediatric Medicine–Allergy Unit, I.R.C.C.S. Children’s Hospital “Bambino Gesù”, Piazza S. Onofrio 4, 00165, Rome, Italy; 2 Rheumatology Research Laboratory, I.R.C.C.S. Children’s Hospital “Bambino Gesù”, Rome, Italy; 3 Department of Paediatric Medicine–Cystic Fibrosis Unit, I.R.C.C.S. Children’s Hospital “Bambino Gesù”, Rome, Italy

**Keywords:** Food allergy, Codfish, Pancreatitis, Challenge, Anaphylaxis

## Abstract

An 8-year-old child during the first year of life manifested severe atopic dermatitis and chronic diarrhea with mucorrhea and rectal bleeding; a fish-free diet was started based on weakly positive skin-prick tests to codfish extract. At the age of 4 years the child began to suffer of recurrent pancreatitis. When he came to our attention for the evaluation of his fish allergy, he was asymptomatic; a weak reactivity to codfish was observed (SPTs: cod, 4 mm, sIgE ImmunoCAP: cod, 1.30kU/l). The food challenge test with cod was negative. When the child ate cod again, within 5 minutes, developed anaphylactic reaction and complained of abdominal pain compatible with pancreatitis (enzyme serum levels risen and parenchymal oedema at ultrasonography), that resolved within 7 days after specific therapy. This case raises two issues: the elimination diet in asymptomatic food allergy on the basis only of SPT and the ethicality of food challenge in gastrointestinal chronic disease.

## Background

Recurrent pancreatitis is characterized by a progressive loss of endocrine and exocrine function. In children pancreatic insufficiency generally develop after several years [1]. Pancreatic damage activated by a food allergy is described only in 7 published reports [2-7].

Here we describe the case of a child who presented an anaphylactic reaction and reactivation of pancreatitis related to the second intake of codfish after specific challenge. This case underscores the possible relation of an elimination diet, started early in life with severe anaphylactic reaction and reactivation of a chronic disease (i.e. pancreatitis). We would alert pediatricians to the risk of food challenge test in a child with a chronic disease in remission.

## Case report

An 8-year-old child came to our Pediatric Allergy Unit to evaluate his cow’s milk (CM) and fish allergy. During the first year of life he manifested severe atopic dermatitis and chronic diarrhea with mucorrhea and rectal bleeding and for this reason he began a CM-free diet.

The association of diarrhea with atopic dermatitis led to the exclusion from diet of foods frequently considered responsible for allergic hypersensitivity (egg, wheat, tomato and peanuts), even though skin-prick tests (SPTs) were negative, and to eliminate fish, that yielded a weakly positive response to extract from codfish. No clinical improvement was observed. At 4 years of age because of persisting anemia and chronic diarrhea, he was treated with mesalazine. Immediately he developed abdominal pain with raised serum amylase and lipase levels. Pancreatitis was diagnosed and two additional episodes occurred while off therapy.

**Figure 1:**
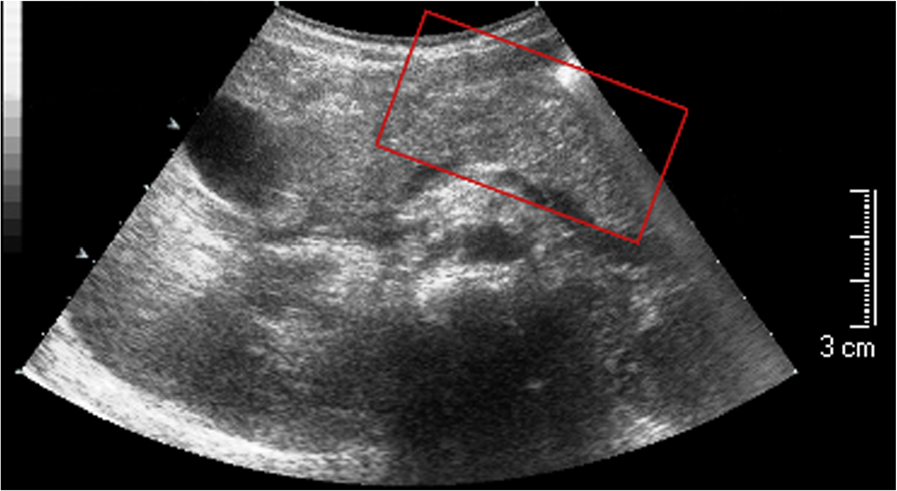
Ultrasound scan showing the pancreas increased in size owing to a large edematous area involving the pancreatic head and tail.

At 8 years of age, when he came to our attention, reactivity to CM was absent (SPTs: CM, negative, histamine, 3 mm; sIgE ImmunoCAP: CM,<0.35kU/l), while weak reactivity to codfish was observed (SPTs: cod, 4 mm, sIgE ImmunoCAP: cod, 1.30kU/l). We reintroduced CM into the diet and then we performed the food challenge test (FCT) with cod that resulted negative. Nevertheless, when he ate cod again, he developed anaphylactic reaction within 5 minutes with rhinitis, asthma, stridor, urticaria and pallor. After resolution (adrenaline im, chorpheniramine iv and hydrocortisone iv), the child complained of abdominal pain; pancreatic enzyme serum levels had risen (alpha-amylase from 104 UI/L to 2559 UI/L). Ultrasonography showed findings compatible with pancreatitis (Figure 1), that resolved within 7 days after specific therapy for pancreatitis (intravenous gabexate mesilate and ranitidine); serum alpha-amylase and lipase levels significantly decreased after 72 hours of treatment (219 UI/L and 114 UI/L, respectively). Since then he has been on fish-free diet and did not experience additional pancreatitis episodes.

## Discussion

Pancreatitis manifesting as an allergic reaction to food is an extremely rare event, particularly when the offensive food is fish and the patient a child. This is the second reported case of pancreatitis related to fish. Previous cases refer to pancreatitis associated with mustard, milk, kiwi and banana [2-7]. The diagnosis of food allergy is essentially clinical [8]. This case raises the problem of prescribing elimination diets in patients with positive *in vitro* diagnostic tests, but asymptomatics [9,10]. In this case codfish, that for the first years of age the child eated without problems, was eliminated by diet only because of the weakly positive sIgE. An accurate clinical history-taking is essential to establish the relationship between food ingestion and allergic symptoms; a positive sIgE alone should not be regarded as sufficient for prescribing a diet.

Before eliminating a food from a child’s diet, the pediatrician should be aware of the possible consequences. Some reports suggest that eliminating a food from the diet could lead to a loss of immunologic tolerance and be responsible for a severe anaphylactic reaction to the next (and often accidental) reintroduction of the suspected food [8-11]. During the first few years of life, despite a weak positive *in vivo* and *in vitro* reaction, our patient tolerated cod. The FCT did after 5 years of fish eliminated diet was negative, but the second introduction of cod determined an anaphylactic reaction and the pancreatitis reactivation. We hypothesize that abstinence from fish was the reason of negative FCT, and the FCT induced the switching on of intolerance to fish with the consequence of an immediated IgE reaction (anaphylactic) that the child never did before, and the reactivation of pancreatitis.

Therefore, this case report raises the question of whether the FCT is ethical in children with gastrointestinal chronic disease.

## Consent

Written informed consent was obtained from the patient’s parents for the publication of this case report and accompaying image. A copy of the written consent is available for review by the Editor-in-Chief of this journal.

## Abbreviations

sIgE, Specific IgE; CM, Cow’s milk; SPT, Skin prick test; FCT, Food challenge test.

## Competing interests

The authors declare that they have no competing interests.

## Authors’ contributions

Each author listed on this short communication has seen and approved the submission of this case report and takes full responsibility for this manuscript. KP: a pediatrician allergist, followed the patient, performed allergy tests, redacted the manuscript and contributed to data analysis; LED: as biologist, performed laboratory exams, collected clinical data and laboratory data into the database, redacted the manuscript and contributed to data analysis; MCA: as allergist, followed the patient in the diagnosis of fish allergy and performed the food challenge test; FA: as gastroenterologist and fellow in cystic fibrosis, followed the patient and performed the ultrasonography image analysis; GC: contributed to manage the study and reviewed the manuscript.
